# Anthracyclines Suppress Both NADPH Oxidase- Dependent and -Independent NETosis in Human Neutrophils

**DOI:** 10.3390/cancers11091328

**Published:** 2019-09-07

**Authors:** Meraj A. Khan, Adam D’Ovidio, Harvard Tran, Nades Palaniyar

**Affiliations:** 1 Program in Translational Medicine, Peter Gilgan Centre for Research and Learning, The Hospital for Sick Children, 686, Bay St., Toronto, ON M5G 0A4, Canada; 2 Department of Laboratory Medicine and Pathobiology, University of Toronto, Toronto, ON M5S 3K1 Canada; 3 Applied Clinical Pharmacology Program, and 4 Institute of Medical Sciences, Faculty of Medicine, University of Toronto, Toronto, ON M5S 3K1, Canada

**Keywords:** drug screening, anthracyclines, DNA-metabolism inhibitors, neutrophil extracellular trap (NET), Nox-dependent and -independent NETosis

## Abstract

Neutrophil extracellular traps (NETs) are cytotoxic DNA-protein complexes that play positive and negative roles in combating infection, inflammation, organ damage, autoimmunity, sepsis and cancer. However, NETosis regulatory effects of most of the clinically used drugs are not clearly established. Several recent studies highlight the relevance of NETs in promoting both cancer cell death and metastasis. Here, we screened the NETosis regulatory ability of 126 compounds belonging to 39 classes of drugs commonly used for treating cancer, blood cell disorders and other diseases. Our studies show that anthracyclines (e.g., epirubicin, daunorubicin, doxorubicin, and idarubicin) consistently suppress both NADPH oxidase-dependent and -independent types of NETosis in human neutrophils, ex vivo. The intercalating property of anthracycline may be enough to alter the transcription initiation and lead NETosis inhibition. Notably, the inhibitory doses of anthracyclines neither suppress the production of reactive oxygen species that are necessary for antimicrobial functions nor induce apoptotic cell death in neutrophils. Therefore, anthracyclines are a major class of drug that suppresses NETosis. The dexrazoxane, a cardioprotective agent, used for limiting the side effects of anthracyclines, neither affect NETosis nor alter the ability of anthracyclines to suppress NETosis. Hence, at correct doses, anthracyclines together with dexrazoxane could be considered as a therapeutic candidate drug for suppressing unwanted NETosis in NET-related diseases.

## 1. Introduction

Neutrophils are the most abundant leukocyte in circulating blood and act as a first line of immune defense [1]. As part of the innate immunity, neutrophils protect the host through phagocytosis, release of cytotoxic molecules, and release of neutrophil extracellular traps (NETs). NET formation (NETosis), was discovered by Takei et al. [2]. It occurs in response to a number of factors, including foreign bodies and changes in pH [3,4,5]. NETs are decondensed chromatin [6] containing antimicrobial peptides which trap and perhaps kill pathogens [7,8,9,10]. Dysregulated NET formation or clearance has been associated with several diseases [11,12,13] and has recently been found to play a role in cancer. NETosis can be induced when neutrophils are in a hypoxic cancer microenvironment [14]. When NETosis is induced without regulation, NETs can damage surrounding cells, tissue and organs. These NETs may also protect tumor cells from the host’s immune system [15]. Moreover, NETs are involved in thrombosis through acting as a scaffold for platelet and red blood cell interactions [15], and can be linked to deep-vein thrombosis in cancer [16]. Thus, further elucidating the pathways of NETosis could have great clinical implications.

Two distinct NETosis pathways have been identified; NADPH oxidase 2 (Nox 2)-dependent [17] and -independent [18,19]. Many different factors, including protein kinases [20,21,22], reactive oxygen species (ROS), neutrophil granular enzymes such as myeloperoxidase (MPO) [23] and the actin cytoskeleton of the neutrophils are involved in regulating various steps of NETosis [24]. Several methods are used for studying NETosis, with more being developed recently by Chicca et al. [25]. Large-scale drug screening is a useful method for identifying the effects of different compounds on specific targets [26]. Many effective antineoplastic compounds were found using a version of this drug screening [27].

Anti-neoplastic compounds affect cell growth and functionality of actively dividing cells, and may exert a different effect on NET formation in terminally differentiated cells such as neutrophils. For instance, anthracyclines (e.g., epirubicin, daunorubicin, doxorubicin, and idarubicin) block DNA and RNA synthesis by intercalating between base pairs [28]. These anthracyclines can also inhibit the activity of topoisomerase-2, a protein that is necessary for transcription and replication of DNA/RNA [29]. Therefore, there may be potential benefits of using compounds used for treating cancer cells in disorders in which NETosis is dysregulated.

In this study, we investigated the effect of cancer chemotherapy drugs on the process of NETosis, while also exploring its mechanisms. Several classes of drugs (39 classes, consisting of 126 drugs) were screened for activity in regulating NETosis, but not one showed an effect as overtly as anthracyclines. We show that the anthracycline class of drugs, such as epirubicin, daunorubicin, doxorubicin, and idarubicin are able to drastically suppress NETosis through both the Nox-dependent and -independent pathways. As specific topoisomerase inhibitors were shown to have little effect, this leads us to hypothesize that the DNA/RNA intercalating activity of anthracyclines is responsible for the effect on NETosis. We also found that these anthracyclines can suppress NET formation without altering ROS production. Furthermore, we found that dexrazoxane, a cardioprotective agent regularly used with anthracyclines [30], does not affect NETosis or the observed anthracycline activity on NETosis. These results reveal a possible mechanism by which anthracyclines are effective in treating those with cancer and dysregulated NET formation in other disease conditions (e.g., life-threatening acute disease such as sepsis). 

## 2. Results

### 2.1. Drug Screening Shows That Anthracyclines Drastically Suppress NETosis

**Figure 1:**
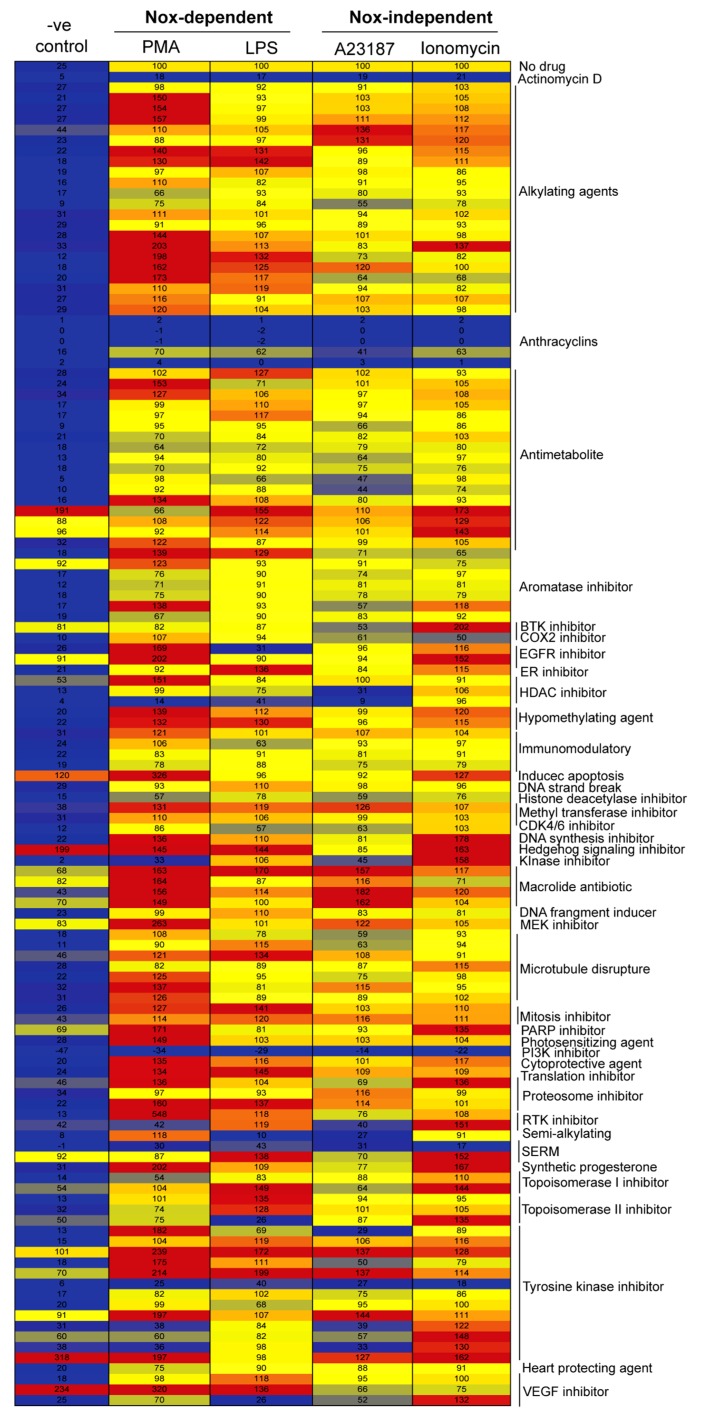
Anthracyclines suppress NETosis induced by both Nox-dependent and -independent pathway agonists. NETosis assays were performed on human neutrophils resuspended with 20 μM of each of the 126 anti-neoplastic compounds with 5 μM Sytox Green fluorescence dye. After one hour, the neutrophils were either unstimulated (-ve control) or stimulated using one of the four agonists PMA (Phorbol 12-myristate 13-acetate) or LPS (Lipopolysaccharides), A23187 or ionomycin). Fluorescence intensities (proxy for DNA release) were measured by a plate reader every 30 min for 4 h. At the last time point (4 h), % DNA release in each conditions were divided by the fluorescent intensity of the cells lysed by Triton-X (Triton lyses the cells and gives maximum fluorescence). Furthermore, the % DNA release (NETosis) in each compound was calculated by considering the 100% DNA release at the last time point (240 min) in respective agonists, as represented in the heatmap. Compounds are grouped by class/mechanism of action. The anthracyclines consistently show less DNA release in each condition.

To determine the effects of the 126 anti-neoplastic compounds on NETosis, we resuspended primary human neutrophils in RPMI (Roswell Park Memorial Institute) medium with 5 μM of Sytox Green fluorescent dye and added 20 μM of each drug (in separate duplicate wells) on a 96-well plate for an hour. These neutrophils were then left untreated (-ve control) or activated by one of the four agonists PMA (Phorbol 12-myristate 13-acetate) or LPS (Lipopolysaccharides) for Nox-dependent NETosis; A23187 or ionomycin for Nox-independent NETosis. Fluorescence emission by Sytox Green was used as a proxy to measure extracellular DNA release, and the signal was measured every 30 min up to 4 h. The kinetics of Sytox Green data was standardized as % DNA release after measuring 100% of the DNA present in the neutrophils by treating the cells (cells lysed) with a detergent Triton-X-100. Further, the total DNA release at 4-h post-activation by agonists (PMA, LPS, A23187 and Ionomycin) treatment were considered as 100% to compare the suppression with drugs in respective agonist conditions. The entire data set was summarized in a heatmap using the data recorded at the 4-h time point (Figure 1). Of interest are the effects of the anthracyclines, as four of the five drugs in this class, epirubicin, daunorubicin, doxorubicin, and idarubicin, greatly suppressed NETosis in the presence of each of the four agonists PMA, LPS, A23187 and ionomycin. Given this remarkable effect of anthracyclines on NETosis, these four compounds were tested further to determine dose-dependency and possible mechanism of action.

### 2.2. Anthracyclines Dose-Dependently Suppress Baseline NETosis

**Figure 2:**
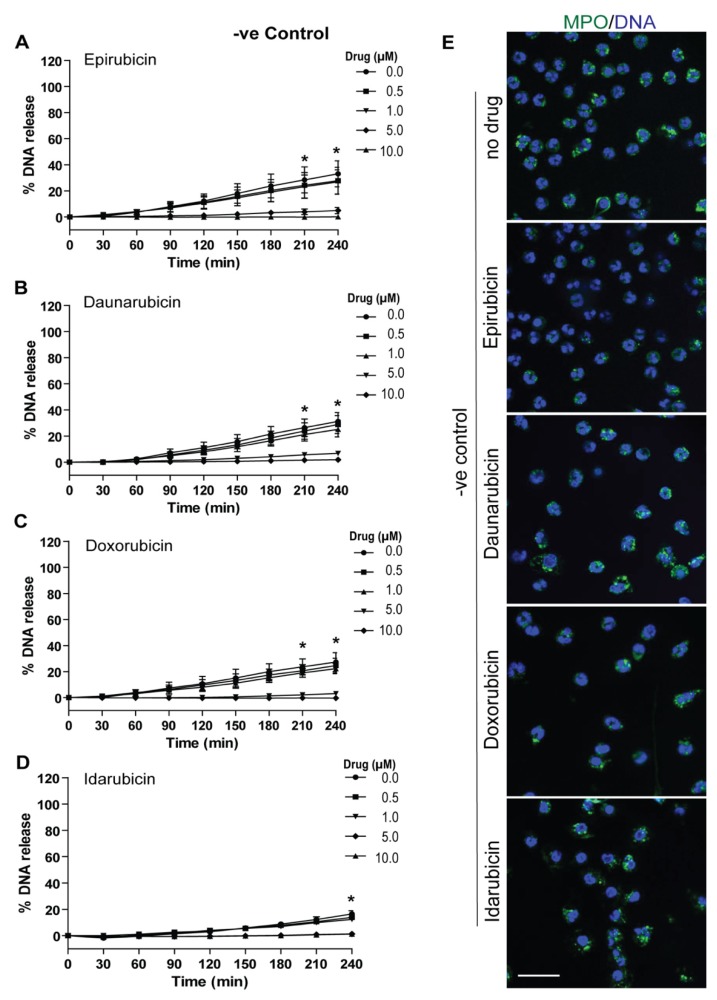
Anthracyclines reduce baseline NETosis in a dose-dependent manner. NETosis assays were performed on human neutrophils resuspended with five concentrations of epirubicin, daunorubicin, doxorubicin, or idarubicin (0.0, 0.5, 1.0, 5.0, and 10.0 μM) and 5 μM Sytox Green fluorescence dye. Neutrophils were unstimulated in order to obtain baseline NETosis information. Fluorescence intensities (proxy for DNA release) were measured by a plate reader every 30 min for 4 h. % DNA release for each condition compared to Triton X-100 sample (100% DNA release) was calculated. Baseline NETosis by (**A**) epirubicin, (**B**) daunorubicin, (**C**) doxorubicin, and (**D**) idarubicin was reduced in a dose-dependent manner (*n* = 3, * *p* < 0.05 between 10.0, 5.0 and 0.0 μM dosage; Two-way ANOVA with Bonferroni’s multiple comparison post-test). (**E**) Confocal microscopy of neutrophils (unstimulated and LPS-treated) were also performed on 5.0 μM samples of each anthracycline. Neutrophils were stained for DNA (blue) and myeloperoxidase (MPO) (green); colocalization of these stains indicates little NET release in each condition (*n* = 3; scale bar, 20 μm).

To further explore the effects of anthracyclines on NETosis, we first resuspended human neutrophils along with 5 µM Sytox Green dye, as before, but with various concentrations of epirubicin, daunorubicin, doxorubicin, and idarubicin (0, 0.5, 1.0, 5.0, and 10.0 μM). Again, fluorescence emissions from the neutrophils were recorded as a proxy for extracellular DNA releases via a plate reader every 30 min for 4 h. The DNA release kinetics show that epirubicin, daunorubicin, doxorubicin and idarubicin dose-dependently suppress baseline NETosis (Figure 2A–D and Figure S1). At the highest dose, 10 μM, % DNA release is nearly at 0% compared to the Triton-X-100 treated samples. To confirm the Sytox-Green-based data, confocal images were obtained for 5 μM drug conditions of each anthracycline after staining the DNA with DAPI and immunostaining for MPO. The intact nuclear morphology and the colocalization of MPO (green) with fewer/less DNA-strings (blue) indicate reduced NETosis in neutrophils treated with these drugs (Figure 2E). The overall immunostained images, confirmed the NETosis suppression by anthracyclines.

### 2.3. Anthracyclines Dose-Dependently Suppress Nox-Dependent NETosis without Affecting ROS Production

**Figure 3:**
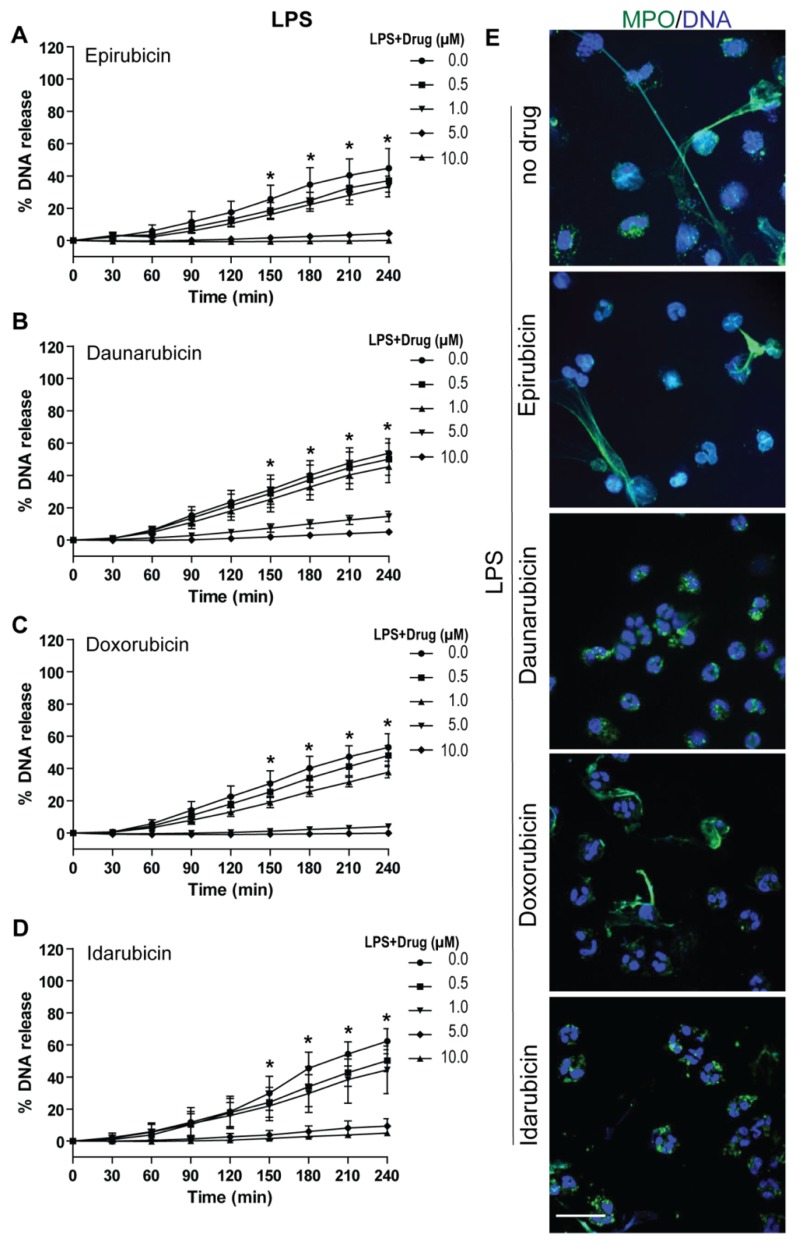
Anthracyclines reduce LPS induced; Nox-dependent NETosis in a dose-dependent manner. NETosis assays were performed in the neutrophils activated with media LPS (25 μg/mL) to induce Nox-dependent NETosis. % DNA release for each condition compared to Triton X-100 (lysed cells, considered as 100% DNA release) was calculated. For (**A**) epirubicin, (**B**) daunorubicin, (**C**) doxorubicin, and (**D**) idarubicin, NETosis was suppressed in a dose-dependent manner (*n* = 3, * *p* < 0.05 between 10.0, 5.0 and 0.0 μM dosage with LPS; Two-way ANOVA with Bonferroni’s multiple comparison post-test). (**E**) Confocal microscopy of neutrophils was also performed on 5 μM samples of each anthracycline. Neutrophils were stained for DNA (blue) and MPO (green); colocalization of these stains shows NETs being released in samples without anthracyclines. With anthracyclines present, the neutrophils are more condensed (*n* = 3; scale bar, 20 μm).

**Figure 4:**
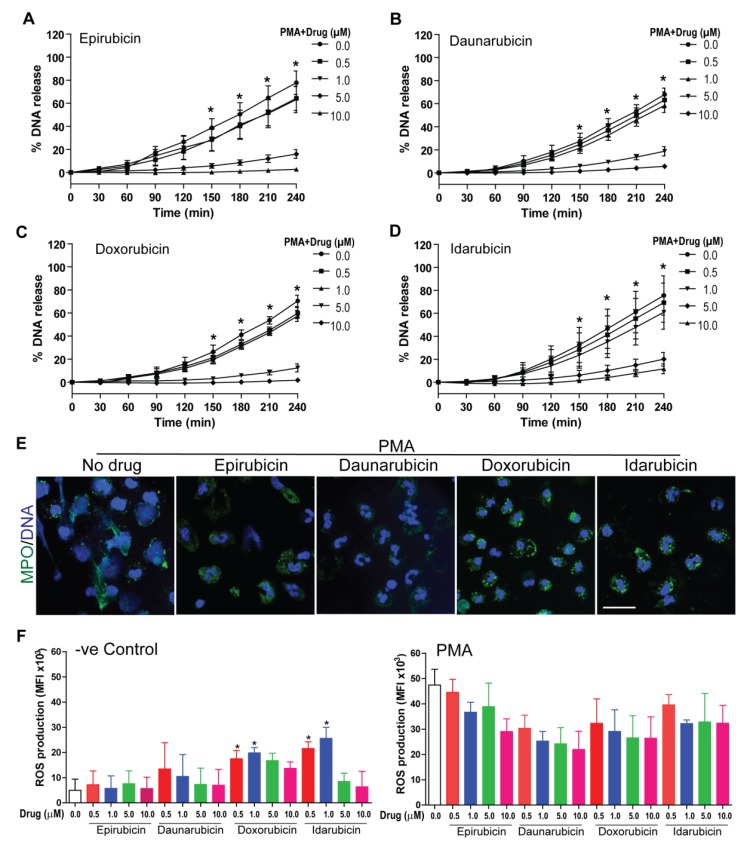
Anthracyclines reduce PMA induced, Nox-dependent NETosis in a dose-dependent manner without affecting reactive oxygen species (ROS) production. NETosis assays were performed but in the neutrophils activated with media PMA (25 nM) to induce Nox-dependent NETosis. The % DNA release for in each condition compared to Triton –X-100 samples (100% DNA release) was calculated. For (**A**) epirubicin, **(B**) daunorubicin, (**C**) doxorubicin, and (**D**) idarubicin, NETosis was suppressed in a dose-dependent manner (*n* = 3, * *p* < 0.05 between 10.0, 5.0 and 0.0 μM dosage with PMA; Two-way ANOVA with Bonferroni’s multiple comparison post-test). (**E**) Confocal microscopy of neutrophils was also performed on 5 μM dosage of each anthracycline drugs. Neutrophils were stained for DNA (blue) and MPO (green); colocalization of these stains shows NETs being released in samples without anthracyclines. With anthracyclines present, the neutrophils are more condensed (*n* = 3; scale bar, 20 μm). (**F**) Neutrophils were loaded with cytosolic ROS indicator DHR123 dye and pre-incubated with epirubicin, daunorubicin, doxorubicin, or idarubicin (0.0, 0.5, 1.0, 5.0, and 10.0 μM) for 1 h. They were then activated with media only (-ve control), or PMA (25 nM). Fluorescence readings (a proxy for ROS production) were taken every 10 min for 30 min. In the media only (-ve control), little ROS was produced. Low-dose doxorubicin and idarubicin increased ROS production. While in the PMA conditions; a substantial amount of ROS was produced. The presence of anthracyclines did not substantially affect ROS production (*n* = 3, * *p* < 0.05; One-way ANOVA with Tukey’s post-test compared to no drug control).

After assessing baseline effects of anthracyclines on NETosis, we examined the effect of epirubicin, daunorubicin, doxorubicin, and idarubicin on the Nox-dependent pathway of NETosis. For these experiments, human neutrophils were resuspended in RPMI medium in the presence of 5 μM Sytox Green dye, as well as Nox-dependent pathway agonists LPS (25 μg/mL) or PMA (25 nM). NETosis, was induced 1 h after the anthracyclines were added to the neutrophils at different concentrations (0.0, 0.5, 1.0, 5.0, and 10.0 μM). The kinetics of DNA release showed that epirubicin, daunorubicin, doxorubicin and idarubicin suppress LPS- (Figure 3A–D and Figure S1) and PMA- (Figure 4A–D and Figure S2) induced NETosis in a dose-dependent manner. For each anthracycline, significant inhibition was detected at 5.0 and 10.0 μM concentrations. 

Again, data was confirmed using confocal images of neutrophils treated with each anthracycline in the presence of agonists. Neutrophil DNA and MPO were stained with DAPI and MPO, respectively. In assessing the morphology of the neutrophils and colocalization of DAPI (blue) and MPO (green), control groups (no anthracycline) are distinct from those with anthracyclines (Figure 3E and Figure 4E). With no anthracycline, neutrophils activated with either LPS or PMA have DNA and MPO present outside of the main cell body, stretching out to form NETs. Neutrophils with an anthracycline present generally appeared more condensed, with less DNA and MPO outside of the cell body, resulting in fewer NETs being formed. Though, in the condition with LPS and epirubicin at 5 μM concentration (Figure 3E), there does seem to be some NETs formation compared to the other anthracycline conditions. The degree of NETosis suppression observed in the images reflects the Sytox green data (Figure 3E and Figure 4E). Taken together, this data suggests that each of the four anthracyclines studied can suppress Nox-dependent NETosis (Figure 3 and Figure 4).

It was important to determine if suppression of NETosis in this manner also suppressed the production of ROS. To assess the impact on ROS generation, we used dihydrorhodamine (DHR-123 dye, which is a fluorescent indicator of ROS. Human neutrophils were incubated with 20 μM of DHR123 for 10 min prior to the addition of epirubicin, daunorubicin, doxorubicin, or idarubicin at 0.0, 0.5, 1, 5, and 10 μM. Then, cells were incubated for 1 h prior to the addition of either only media (-ve control), or PMA (an inducer of ROS production). After activation, fluorescence—a proxy of ROS generation—was measured every 10 min for up to 30 min by a microplate reader (Figure 4F). The control conditions generally showed minimal ROS production at the last time point. However, there was some baseline ROS production compared to -ve control in the presence of lower concentrations of (0.5 and 1.0 µM) doxorubicin and idarubicin. The PMA samples showed an increased amount of ROS production compared to the control, and this production was not suppressed by the presence of any anthracycline (Figure 4F). Therefore, at some concentrations, certain anthracyclines may increase ROS production, none of the four anthracyclines significantly increased ROS production during agonist-mediated activation of neutrophils.

### 2.4. Anthracyclines Dose-Dependently Suppress Nox-Independent NETosis

**Figure 5:**
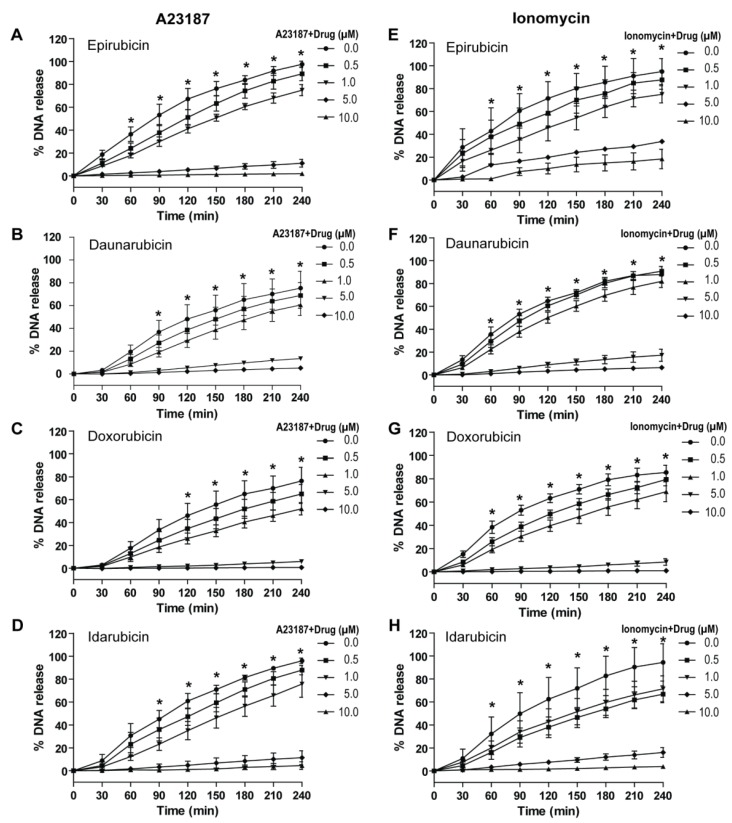
Anthracyclines reduce A23187- and Ionomycin- induced, Nox-independent NETosis in a dose-dependent manner. Neutrophils were activated with either A23187 (4 μM) or ionomycin (1 μM) to induce Nox-independent NETosis. NETosis kinetics estimated as earlier. A23187 and ionomycin induced NETosis suppressed by (**A**,**E**) epirubicin, (**B**,**F**) daunorubicin, (**C**,**G**) doxorubicin, and (**D**,**H**) idarubicin in a dose-dependent manner, respectively, (*n* = 3, * *p* < 0.05 between 10.0, 5.0 and 0.0 μM dosage with A23187 or ionomycin; Two-way ANOVA with Bonferroni’s multiple-comparison post-test).

**Figure 6:**
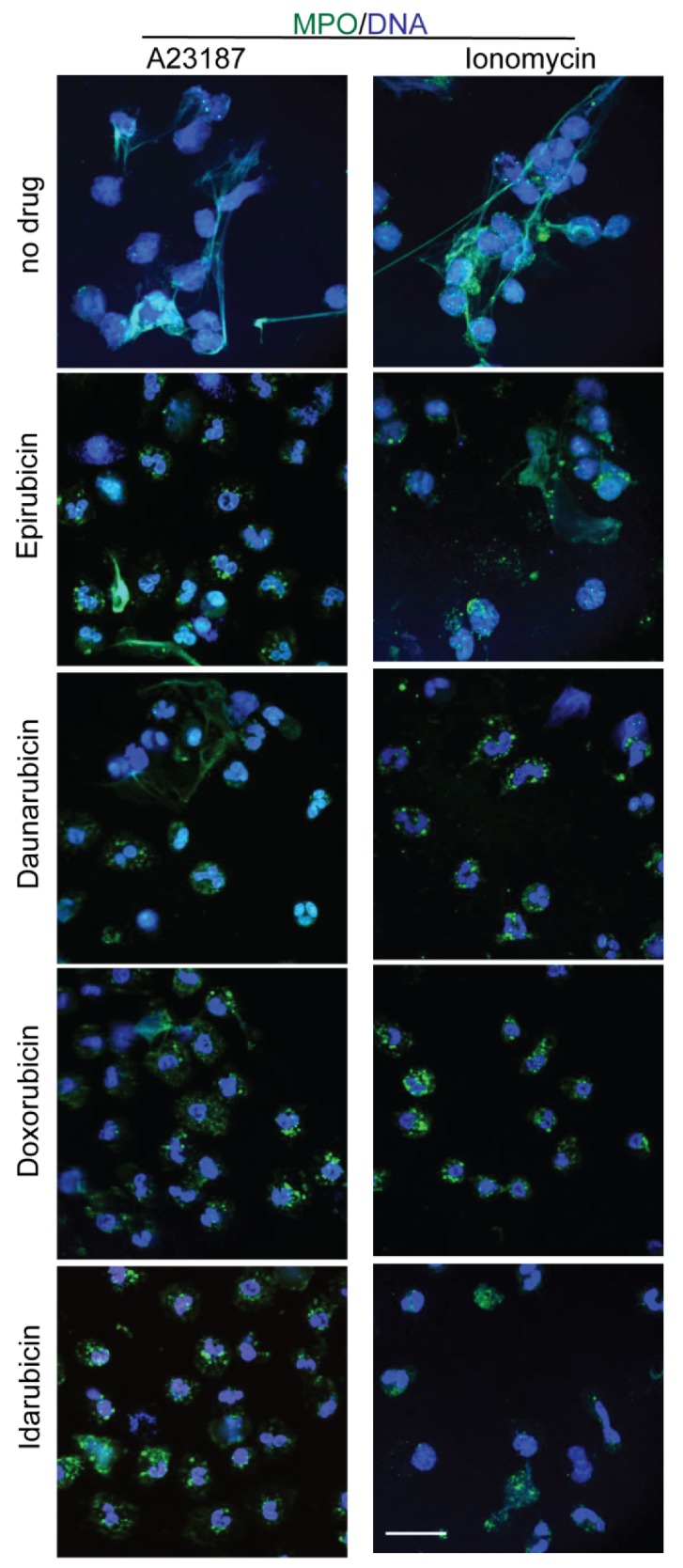
Confocal images confirm the A23187- and Ionomycin-induced NETosis suppression by anthracyclines. Fluorescence staining and confocal microscopy of neutrophils was performed on 5 μM samples of each anthracycline with and without A23187 and ionomycin. Neutrophils were stained for DNA (blue) and MPO (green); colocalization of these stains shows NETs being released in samples without anthracyclines. With anthracyclines present, the neutrophil nuclei are more condensed and significantly fewer NETs release (*n* = 3; scale bar, 20 μm).

After assessing the Nox-dependent pathway, it was important to study the regulatory effect of anthracyclines in the Nox-independent pathway of NETosis. As before, human neutrophils were used in a NETosis assay with epirubicin, daunorubicin, doxorubicin, and idarubicin. The neutrophils were plated in the presence of 5 μM Sytox Green dye, an anthracycline, and a Nox-independent agonist (A23187, 4 μM or bacterial ionomycin, 1 μM). NETosis was induced 1 h after the anthracyclines were added to the neutrophils at several concentrations (0.5, 1.0, 5.0, and 10.0 μM). To determine the kinetics, fluorescence emissions from the neutrophils were recorded every 30 min for 4 h. The kinetics for DNA release show that epirubicin, daunorubicin, doxorubicin and idarubicin suppress A23187 and ionomycin induced NET production in a dose-dependent manner (Figure 5A–H and Figure S3). Each anthracycline reduced NET production significantly at 5.0 and 10.0 μM concentrations. Furthermore, the NETosis data was confirmed using confocal images (Figure 6). At 5 μM conditions of each anthracycline, neutrophils were stained for DNA (DAPI, blue) and MPO (green). Based on the morphology of the neutrophils and colocalization of DAPI and MPO, the control group (no anthracycline) is distinctly different compared to those with anthracyclines present. Neutrophils activated with either A23187 or ionomycin have distinctly more extracellular DNA and MPO, extending from the decondensed nuclei as NETs. As with Nox-dependent NETosis, the presence of anthracyclines resulted in a more condensed morphology, like the control samples discussed. This data suggested that the four anthracyclines can suppress Nox-independent NETosis.

Furthermore, to understand the mechanistic aspect of the anthracyclines, we used the topoisomerase-2 inhibitor (Banoxantrone dihydrochloride) and perform the NETosis kinetics in the presence and absence of both NOX-dependent (PMA, and LPS) and NOX-independent (A23187 and Ionomycin) agonists. We did not observe the significant suppression of NETosis by topoisomerase-2 inhibitor (also, see Figure 1). Anthracyclines can inhibit topoisomerase-2 but they also have many other effects in neutrophils during NETosis. The intercalating property of anthracycline may be enough to stop the transcription initiation and exert NETosis inhibition.

### 2.5. Dexrazoxane, a Cardioprotective Agent, Does Not Alter the Suppressive Effect of Anthracyclines on NETosis

**Figure 7:**
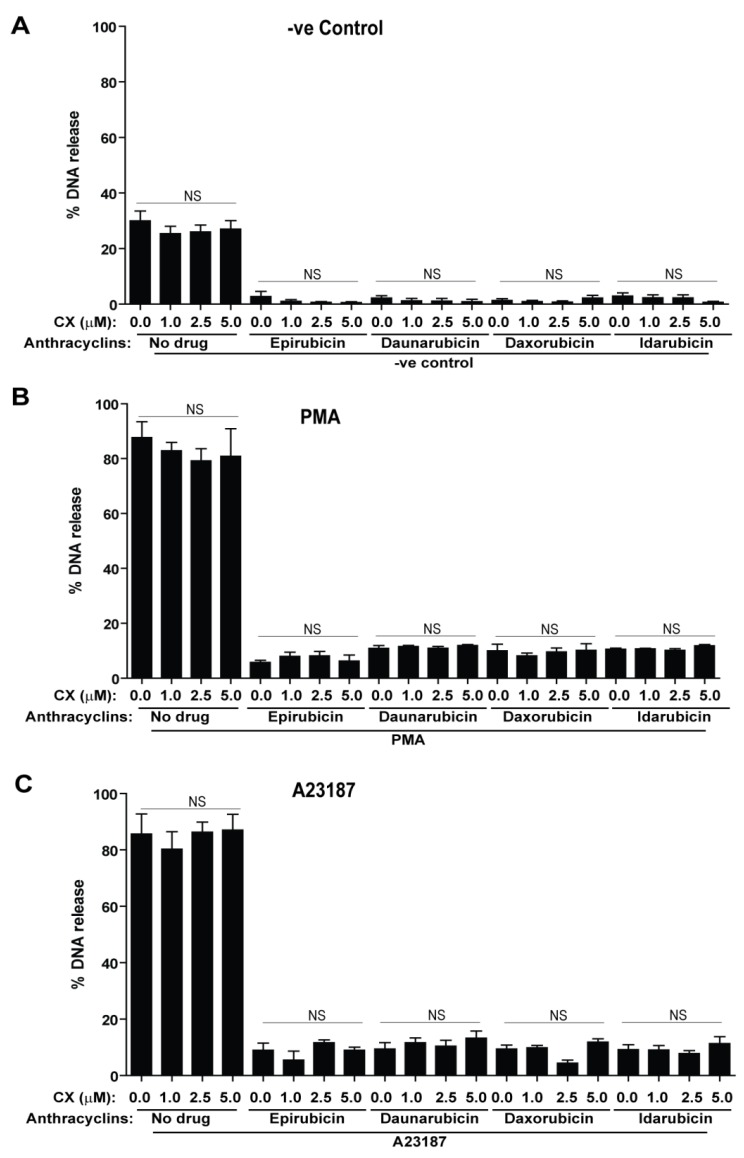
Dexrazoxane does not induce NETosis, nor alter the effect of anthracyclines on NETosis and anthracycline-mediated NETosis suppression does not induce apoptosis. NETosis assays were performed on human neutrophils that were plated with one concentration (5 μM) of epirubicin, daunorubicin, doxorubicin, or idarubicin, dexrazoxane (0.0, 1.0, 2.5, 5.0 μM) and 5 μM Sytox Green fluorescence dye. NETosis was induced using either media (-ve control), PMA (25 nM), or A23187 (4 μM). Fluorescence intensities (proxy for DNA release) were measured by a plate reader every 30 min for 4 h. % DNA release for each condition compared to Triton –X-100 sample (100% DNA release) was calculated. Bar charts were created to compare % DNA release. For (**A**), dexrazoxane alone did not affect the production of NETs as % DNA release is similar to control conditions. For (**B**,**C**), it is shown that dexrazoxane did not affect how anthracyclines suppress NETosis via either pathway (*n* = 3, * *p* < 0.05; One-way ANOVA with Tukey’s post-test compared to 0.0 μM dosage of control drug control).

Anthracyclines, like many anti-neoplastic agents, are potentially harmful to the human body in addition to their obvious benefits [31]. In particular, anthracyclines are known to be cardiotoxic, and their cardiotoxicity is in fact a dose-limiting factor when they are being used for cancer treatment. To combat this toxicity, dexrazoxane is typically used as a cardioprotective agent in conjunction with anthracyclines. It is unknown whether this adjuvant compound affects NETosis, and thus we determined if dexrazoxane could suppress or induce NETosis, as well as if it could affect the ability of anthracyclines to suppress NETosis. For these experiments, human neutrophils were used in a NETosis assay with epirubicin, daunorubicin, doxorubicin, or idarubicin (5 μM each), and/or dexrazoxane (0.0, 1.0, 2.5, 5.0 μM). In addition, 5 μM Sytox Green dye was added, and after 1 h, neutrophils were activated with either PMA (25 nM), or A23187 (4 μM). Using fluorescence emissions as a proxy for DNA release, readings were taken every 30 min for 4 h. Total DNA release at the last time point data show that dexrazoxane does not induce NETosis (Figure 7A), nor does it impede with the capabilities of the tested anthracyclines to suppress NETosis, in PMA- and A23187-treated neutrophils (Figure 7B,C).

### 2.6. Doses of Anthracyclines That Fully Suppress NETosis Do Not Induce Apoptosis

**Figure 8:**
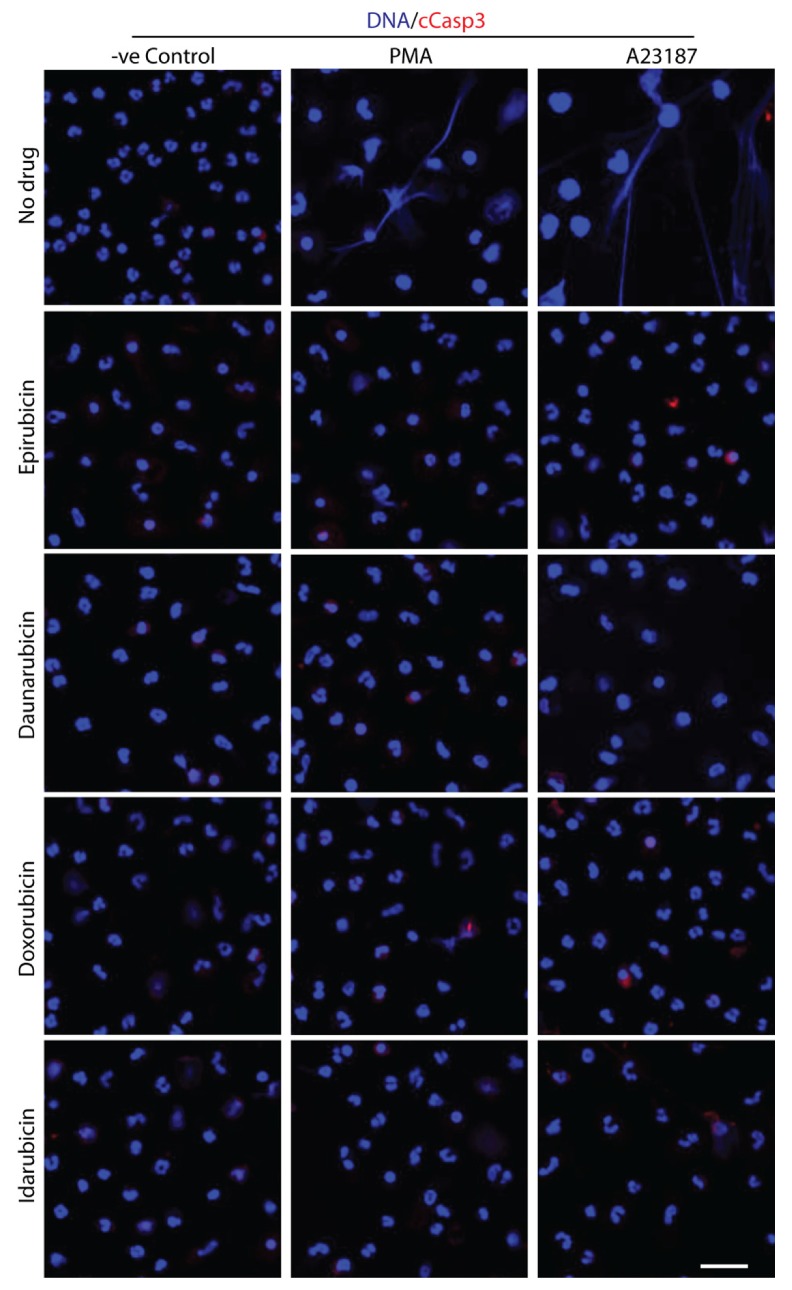
Anthracycline-mediated NETosis suppression does not induce apoptosis. Neutrophils were incubated with anthracyclines and then induced to produce NETs using either media only, PMA (25 nM) or A23187 (4 μM) and then fixed to 8-chamber slides. cCasp-3 and DNA were stained with anti-cleaved cCasp-3 rabbit primary antibody/goat anti-rabbit secondary antibody (red) and DAPI (blue), respectively. For –ve control (media only), PMA, and A23187 conditions, cCasp-3 release was not increased compared to media control (*n* = 3; scale bar 20 μM). The counting data of NETosis and apoptosis is represented in Figure S4.

Apoptosis is another process of programmed cell death, but it occurs through different mechanisms compared to NETosis [32]. While apoptosis serves many functions, from embryogenesis to fighting off disease [33], inducing it out of turn can be detrimental. Therefore, it was important to analyze the effect that the higher concentrations of epirubicin, daunorubicin, doxorubicin, and idarubicin (5.0 μM) have on apoptosis. Neutrophils that were exposed to anthracyclines and then activated with PMA or A23187 were fixed onto chamber slides. Cleaved caspase-3 (cCasp-3), a typical marker for apoptosis [20], was stained on these slides using anti-cleaved cCasp-3 rabbit primary antibody and goat anti-rabbit secondary antibody at a dilution of 1:1000. DAPI was also used to stain DNA present. These slides were imaged by confocal microscopy. As cells undergo apoptosis, the nuclei condense, and cCasp-3 is generated [20]. The percentage counting of the normal cells, NETosed (DNA decondensation and DNA-NET fibers, taken together) and apoptosed cells data showed that the neither of the drugs induces apoptosis during the suppression of NETosis in unstimulated (control), PMA- and A23187-activated neutrophils (Figure 8 and Figure S4).

## 3. Discussion

**Figure 9:**
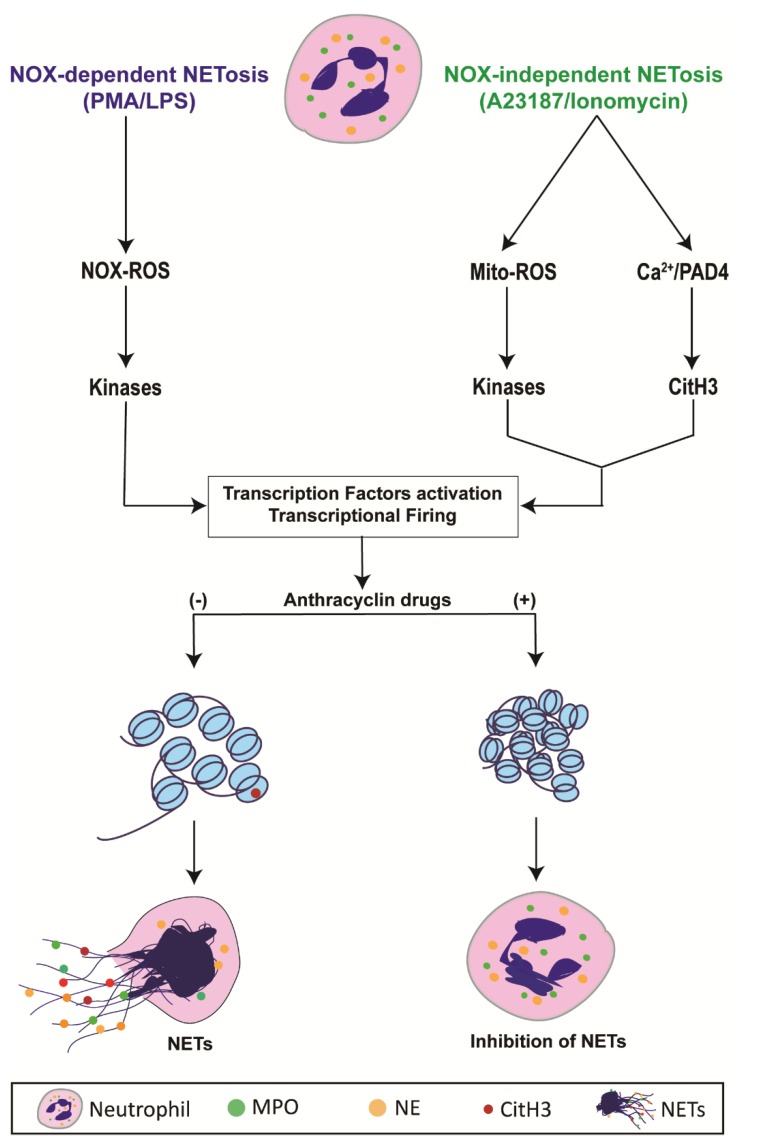
Diagrammatic illustration shows NETosis regulation by anthracyclines. Neutrophils activated either with NOX-dependent (agonists; PMA, LPS) or -independent (agonists; A23187, Ionomycin) induces NETs release through the activation of respective ROS, kinases, and transcriptional firing. Anthracycline compounds intercalate between DNA base pairs and stop the unwinding, decondensation and transcriptional firing to stop the NETosis. Since this step is downstream of the ROS, these drugs are helpful in suppressing the NETs release with maintaining the neutrophils ROS generation capacity.

There have been many studies regarding the mechanism of NETosis in recent years. As the mechanism is uncovered, more treatments could be formulated to combat conditions in which NETosis is dysregulated, such as cancer [14,34]. Through investigating anti-neoplastic compounds and their effect on NETosis, we found that epirubicin, daunorubicin, doxorubicin, and idarubicin (anthracycline drugs) can suppress NETosis, which was induced via the Nox-dependent or -independent pathway. Through conducting a drug screening of 126 anti-neoplastic compounds (Figure 1), these anthracyclines stood out in their suppression of NETosis. We further investigated these four compounds to determine their dose-dependent effects and confirmed that they act in a dose-dependent fashion, inhibiting NET production to a higher degree at increased concentrations. Moreover, we found that these compounds did not significantly suppress agonist-induced ROS production, which is known to be necessary for Nox-dependent NETosis [35]. These compounds could be used as NETosis-suppressive drugs while maintaining the ROS generation capability of neutrophils (Figure 9). 

Anthracyclines hamper chromatin/DNA unwinding by interacting with DNA topoisomerase-2, a necessary complex for DNA replication and transcription [36]. This information, in tandem with our recent transcriptomic analyses [37], suggest anthracyclines suppress NETosis by blocking transcriptional firing. Neutrophil viability was assessed through apoptosis experiments, and we confirmed that anthracyclines were not causing the cells to undergo apoptosis instead of NETosis, as they are known to cause some cardiotoxicity [38]. Dexrazoxane is used clinically to prevent this toxicity in cancer patients receiving anthracyclines [38]. Our experiments showed that dexrazoxane does not affect NETosis, nor does it affect the ability of anthracyclines to suppress NETosis. Together, our data suggests that anthracyclines are potential candidates for further studies regarding suppression of NETosis as they are both safe and effective (Figure 7). DNase can be used for degrading the NETs (in case of excess or dysregulated NET formation), while anthracyclines for suppressing the NETs formation by neutrophils and restoring neutrophils functionality of producing ROS.

A paper by Swystun, Mukherjee, and Liaw (2011) investigated how epirubicin and doxorubicin on their own affect cell-free DNA release and coagulation [39]. One of the assays in that study showed that these drugs increased cell-free DNA, whereas another assay showed that they inhibited the release of DNA-histone complex from neutrophils. These findings contradict with each other, but it appears that the anthracyclines could have been suppressing NETosis. Because NETosis is not defined as cell-free DNA, rather a complex mesh of DNA-histone entangled with granular proteins. Hence, the conclusion about the effect of these drugs on NETosis was unclear.

It is well known that NETosis can occur via two different pathways, Nox-dependent or Nox-independent, based on what agonist or foreign body is present [40]. We have previously investigated other compounds inhibiting NETosis, such as actinomycin-D [37], but a large suite of anti-neoplastic compounds has not been investigated. In performing a large-scale screen, most anthracyclines were identified as having strong suppressive effects on NETosis (Figure 1). While other compounds showed some suppressive or even inductive effects on NETosis, they were not pursued in this study as these anthracyclines showed greater potential for further study. From this point, it was necessary to determine if these four anthracyclines affected NETosis in a dose-dependent manner. In general, dose-dependency is more highly favored in the realm of therapeutic compounds; having options for providing a patient with a higher dose without achieving toxicity, or vice versa, can be incredibly important clinically [41]. Using several known agonists of NETosis, LPS, PMA, A23187, and ionomycin, each anthracycline was found to dose-dependently suppress agonist-induced NETosis (Figure 2, Figure 3, Figure 4, Figure 5 and Figure 6).

Previous work has established that PMA and LPS induce Nox-dependent NETosis [42,43,44], while A23187 and bacterial ionomycin induce Nox-independent NETosis [45,46]. The Nox-dependent pathway depends on ROS production through Nox, and it has been previously shown that inhibiting Nox blocks NET production through this mechanism [19,47]. While being able to inhibit Nox-independent NETosis, as well as Nox-dependent NETosis, is a clue towards the mechanism of anthracycline suppression, their effect on Nox and ROS production was further investigated. Anthracyclines did not significantly affect ROS production when PMA was used as an agonist (Figure 4F). However, it is of note that in a control condition, doxorubicin and idarubicin increased ROS production in un-agonized neutrophils at 0.5 and 1 µM, but not at 5 or 10 μM (Figure 4F). It is not unusual for anthracyclines to cause ROS production, as both doxorubicin and idarubicin have been found to produce ROS at certain concentrations, as oxidative stress was originally thought to be the primary mechanism for anthracycline-induced cancer cell toxicity [48]. 

It is unexpected that this ROS production occurred only at lower concentrations, in addition to the fact that neither daunorubicin nor epirubicin had the same outcome. Moreover, studies have shown that doxorubicin may induce Nox-mediated oxidative stress [49]. Chromatin decondensation and initial transcription constitute one of the key final steps in both Nox-dependent and -independent NETosis. Drugs or compounds that stop these targets would also suppress NETosis without affecting ROS production. Since agonists known for producing ROS have been used along with the drugs that work at chromatin level, the NETosis suppression has been observed, along with the unaltered ROS generation. Additional information regarding the activity of Nox in neutrophils in the presence of anthracyclines may uncover more information in this regard. Keeping this in mind, it does seem that anthracycline suppression of NETosis occurs downstream of ROS production.

Anthracyclines can be toxic to human cells along with their cancerous targets. The exact mechanism of anthracycline toxicity is still being investigated and recent reports are focused on their ability to affect DNA torsion in cells, which can lead to nucleosome destabilization and subsequent cell death [50]. This toxicity generally leads to apoptosis in cells [50]. As discussed, the drug dexrazoxane is typically used in conjunction with doxorubicin as a protective agent. It effectively prevents the cardiotoxicity that is generally caused through the use of anthracyclines [31]. Therefore, it was of interest in this study to determine its effects on NETosis, both alone and as an adjuvant to doxorubicin and the other three anthracyclines. In the initial drug screening, dexrazoxane had no significant effect on either pathway of NETosis (Figure 1). Additional Sytox Green assays were conducted using dexrazoxane and the anthracyclines as before (Figure 7A–C), showing that the dexrazoxane did not increase or decrease the suppressive ability of the anthracyclines used in this project.

Anthracyclines typically cause significant cancer cell death through apoptotic mechanisms. Therefore, there is potential that anthracyclines could cause neutrophils to undergo apoptosis instead of NETosis. In apoptosis, the structure of DNA and the molecules released into extracellular space are very different than in NETosis [51], as per the activation pathway. Immunohistochemistry confirmed that neutrophils, in the presence of anthracyclines, were undergoing limited NETosis, and no apoptosis (Figure 8 and Figure S4). If apoptosis were occurring, DNA would have appeared condensed, and cCasp-3 (a marker of apoptosis) would have been present in high amounts [20]. Therefore, anthracyclines, at the concentrations used in this study, are reasonably safe for further investigations with neutrophil—specifically NETosis—functioning.

It was hypothesized that compounds that affect DNA replication or transcription would be most effective at preventing NET production. Anthracyclines are inhibitors of topoisomerase-2. Topoisomerases have many biological functions, one of which is to unwind DNA during replication and transcription [52]. In addition, anthracyclines are able to intercalate in DNA and RNA, inhibiting their function [53]. We did not observe the significant suppression of NETosis by topoisomerase-2 inhibitor. We have reported earlier that the transcription initiation, but not full transcription of genes per se, helps to drive both types of NETosis [37]. Therefore, using only topoisomerase-2 inhibitor is not enough to suppress the NETosis. As discussed, it is likely that transcription plays an important role in NETosis. For example, Keshariet et al., determined that ERK (extracellular signal-regulated kinases) plays an integral role in the mediation of PMA-induced NET release [22]. ERK is known to activate several transcription factors and kinases once it is phosphorylated. Recently, it was confirmed that transcription plays an important role in NETosis, as directly blocking transcription without affecting ROS production halts the release of NETs [37]. The addition of the anthracyclines may be blocking this downstream activation and, therefore, inhibiting NETosis as shown in diagrammatic illustration (Figure 9). Doxorubicin and daunorubicin have been shown to block transcription through inhibitory interactions with RNA polymerase II [54,55], and may be acting in such a manner to slow NET production. It is intriguing that topoisomerase inhibitors such as topotecan [56], nor alkylating agents (which intercalate within DNA) such as dacarbazine [57], had no effect on NETosis (Figure 1). Perhaps it is the combination of activity that anthracyclines have that enables them to effectively inhibit NET production.

The process of NETosis, while mostly beneficial, has been implicated in several disorders. It has been hypothesized that dysfunctional NET formation may be present in periodontitis, an infectious-inflammatory disease of the gums [58]. Abnormal amounts of neutrophils are present in the gums of those with this condition, and high levels of ROS production have been detected [59]. NET production, as per the Nox-dependent pathway, relies on ROS generation, and may be occurring during periodontitis. Dysregulated NETosis may also be associated with rheumatoid arthritis (RA) [12]. In those with RA, neutrophils are more likely to produce NETs. Furthermore, it is reported that the severity of RA increases as more NETs are produced [60]. Tumor-induced NETosis has even been identified as a risk factor for organ failure in cancer [61]. With possible roles in multiple conditions apart from periodontitis, RA, and cancer, discovering an effective and safe method of inhibiting NETosis would be clinically advantageous. Anthracyclines, given at a safe dose, could be effective treatments for the NETotic dysregulation in these conditions. Dexrazoxane and, potentially, granulocyte colony stimulating factor, a compound known to reduce neutropenia which is caused by anthracyclines [62], could be useful as adjuvants in this potential therapeutic scenario. It is likely that anthracyclines are inhibiting NETosis via topoisomerase-2 inhibitions and intercalation within DNA, hampering the processes of replication and transcription. Further study of anthracyclines, including those not studied here, in conditions associated with dysregulated NET production such as sepsis, RA and cystic fibrosis [12,63,64] are warranted based on these results. Collectively, data suggest that these compounds would be useful in suppressing the NETosis, while keeping the ROS generation capability of neutrophils. 

## 4. Materials and Methods 

### 4.1. Research Ethics Board Approval

The ethics committee of The Hospital for Sick Children (Sick Kids), Toronto, approved the study protocol for the use of human blood samples. Each procedure, including healthy human volunteer recruitment for blood donation, was performed in agreement with the guidelines set by the ethics committee. All volunteers who participated in this study gave their signed consent prior to blood donation. 

### 4.2. Reagents

SYTOX^®^ Green Nucleic Acid Stain dye and DHR123 were acquired from Molecular Probes (Thermo Fisher Scientific, Waltham, MA, USA). All buffers, agonists, and other reagents were acquired from Sigma Aldrich unless stated otherwise. For these experiments, the standard medium was RPMI 1640 medium (Invitrogen, Carlsbad, CA, USA) that was supplemented with 10 mM HEPES buffer. All anti-neoplastic compounds used were graciously donated to us by the National Cancer Institute. We received 126 compounds from the “Approved Oncology Drugs” set. It consisted of 2 plates containing 20 μL of a 10 mM stocks (in DMSO) of each compound. 

### 4.3. Initial Screening Dosage Selection

It is important to make sure that drugs work and are safe to the cell system. To begin the screening, we want to make sure to use a dosage that would be enough to inhibit NETosis by all the drugs. The IC50 values of the different drugs comprise a wide range based on the different cell type and duration (for example, anthracycline; 0.35–6.5 µM, alkylating agents; ~10 µM, protein kinase inhibitors; ~8–10 µM) [65,66]. Previous reports related to drug screening strategies, made some consideration for the selection of compound/drug screening dosages [67,68]. The cell-based compounds screening assays for hit discovery are typically run at 1–20 µM compound concentration. This concentration may vary based on the type of cells and duration of the drug treatment and a dosage of up to 40 µM can be used. Therefore, we have selected a consistent single dosage (20 µM) for all the drugs.

### 4.4. Neutrophil Isolation

Peripheral blood was collected from healthy donors in K2 EDTA blood collection tubes (Becton, Dickinson and Co., Franklin Lakes, NJ, USA). Neutrophils were then isolated from the whole blood using PolymorphPrep (Axis-Shield, Dundee, UK) based on the manufacturer’s instructions, with slight modifications as reported previously [18,20,21,37]. In brief, equal volumes of blood were layered over the PolymorphPrep and spun for 35 min at 600× *g*, 25 °C to separate the band of neutrophils. The neutrophil band was washed, and then lysis of red blood cells was performed with a hypotonic solution [0.2% (*w*/*v*) NaCl] for 30 s followed by the addition of an equal volume of 1.6% (*w*/*v*) NaCl solution with HEPES buffer (20 mM, pH 7.2) to buffer the solution and ensure it was isotonic. Next, the isolated neutrophils were resuspended in RPMI medium supplemented with HEPES buffer (10 mM, pH 7.2). Neutrophil counting and a viability check were carried out by using trypan blue and a hemocytometer. Moreover, Cytospin preparation and imaging were used to confirm the purity of neutrophils. In order to be used in experiments, neutrophil preparations must be >95%–98% live and pure. Multiple donors were used to acquire sufficient replicates for each experiment.

### 4.5. Sytox Green- NETosis Assay

For microplate reader assays, cells were seeded at 50,000 cells per well in a 96-well plate in the culture media in the presence of Sytox Green cell-impermeable nucleic acid stain at 5 µM. Sytox Green binds to extracellular DNA and fluoresces. Cells were pre-incubated with drugs for 1 h before the activation of NETosis. During the initial screening of all compounds, drugs were used at 20 µM. For the validation experiments using the four anthracyclines (idarubicin, doxorubicin, daunorubicin, and epirubicin), a serial dilution was utilized to obtain concentrations of 0.5–10.0 μM for anthracyclines. Dexrazoxane was used as an adjuvant to the anthracyclines in different concentrations (0.0, 1.0, 2.5, 5.0 μM). NETosis was induced using A23187 (calcium ionophore) (4 μM), PMA (25 nM), bacterial ionomycin (1 μM), and LPS (25 μg/mL). Fluorescence was measured using a POLARstar OMEGA fluorescence microplate reader (504 nM excitation and 523 nM emission, BMG Labtech) at 30 min intervals up to 4 h following induction of NETosis. A total of 900 data points per well were scanned at each time point to quantify the fluorescence intensity. Fluorescence values of cells lysed with 0.5% (*v*/*v*) Triton X-100 were used to determine total DNA (100% DNA) present in neutrophils for each experiment. In order to calculate the percentage of NETosis in each condition, the green fluorescence at time 0 min was subtracted from the fluorescence at each time point. These new values were then divided by the fluorescence values of cells lysed with 0.5% (*v*/*v*) Triton X-100.

In a typical NETosis kinetics assay, we used –ve control (no agonist; only media control; to assess the base line NETosis), agonists (known NETosis inducer; in presence and absence of drugs) and +ve control (0.5% triton-X-100; lysed the cells and the total DNA fluorescence considered as 100%). The % DNA release in each condition has been calculated by considering the +ve control fluorescence as 100%. These −ve and +ve controls have been used in each experiment, including with and without anthracyclines. Each condition was tested in duplicate. Experimental replicates (n-values; donors) of independent experiments were reported in the figure captions.

### 4.6. DHR123- ROS Assay

DHR123 dye (ThermoFisher Scientific, Waltham, MA USA) was used to measure intracellular ROS production. Primary neutrophils (10^6^ cells/mL) were incubated with 20 μM DHR123 for 10 min as per the manufacturer’s instructions, with slight modifications as reported previously [18,20,69]. The cells were washed to remove extracellular DHR123 dye and cells were re-suspended in fresh RPMI media. Next, 50 μL of cell suspension was plated in a well within a 96-well plate (50,000 cells per well). These cells were pre-incubated with each of the four anthracyclines (doxorubicin, daunorubicin, idarubicin and epirubicin) for 1 h prior to activation as in the NETosis Assays discussed above. Cells were activated with media (negative control), PMA, or A23187 for an additional 30 min. To assess the kinetics of the ROS generation in various conditions, the fluorescence generated was measured every 10 min by an Omega fluorescence microplate reader (900 data points per well). Each condition was tested in duplicate. Experimental replicates (n-values; donors) of independent experiments were reports in figure captions.

### 4.7. Immunofluorescence Confocal Microscopy

Cells that had been induced to produce NETs were fixed with paraformaldehyde (4% (*w*/*v*) for 10 min; 2% (*w*/*v*) onto 8-chamber slides (BD Falcon, New York, NY, USA) overnight, and immunostained with several NET markers. For MPO staining, mouse anti-myeloperoxidase antibody (ab25989, Abcam, Cambridge, MA, USA) at 1:500 dilution was used (with secondary antibody conjugated with a green fluorescence Alexafluor 488 dye; 1:2000 dilution; Thermo Fisher Scientific, Waltham, MA USA)) and DAPI (1:1000 dilution) was used to stain DNA. Eight-well chamber slides (Falcon culture slides) were used to obtain high-resolution images. Following immunostaining, the slides were mounted with anti-fade fluorescent mounting medium (Dako, Santa Clara, CA, USA) and glass cover slips (Fisher Scientific, Markham, ON, Canada)). NETosis was identified by MPO to NET DNA colocalization by an immunofluorescence confocal microscopy (Olympus IX81 inverted fluorescence microscope equipped with a Hamamatsu C9100-13 back-thinned EM-CCD camera and Yokogawa CSU × 1 spinning disk confocal scan head). The confocal images were taken at 40× magnification with 1.35× objective, and processed by Volocity software (Version 6.3, Cell Imaging Perkin-Elmer, Quorum Technologies Inc., Puslinch, ON, Canada).

### 4.8. Confocal Imaging of Apoptosis

Neutrophils were prepared for immunostaining of apoptosis markers using the same procedure as described previously [20,51] and in the section “Immunofluorescence Confocal Microscopy” by using a 5 μM drug concentration. For cCasp-3 staining, anti-cleaved cCasp-3 rabbit primary antibody (Asp175, Cell Signaling) and goat anti-rabbit secondary antibody (Alexa Fluor 555, A21428, Life Technologies, Carlsbad, CA, USA) at 1:1000 dilutions were used. Moreover, these immunostained slides were imaged via confocal microscopy as formerly described.

### 4.9. Statistical Analyses

GraphPad Prism statistical analysis software (Version 5.0a) was used to perform all statistical analysis. When comparing two groups, a Student’s *t*-test was used, and for more than two groups, ANOVA with Bonferroni’s posttest or Dunnett’s test was used where appropriate. Technical repeats and applied statistics are mentioned in each figure caption. A *p*-value of ≤0.05 was considered statistically significant. All data are presented as mean ± SEM.

## 5. Conclusions

Currently, there are no compounds known to specifically inhibit NETosis. The purpose of this study was to uncover possible anti-NETosis compounds from a pool of currently available drugs (epirubicin, daunorubicin, doxorubicin, or idarubicin). Through a large-scale drug screening, anthracyclines were identified as inhibitors of NETosis, while maintaining ROS generation capacity and not causing neutrophil cell death. These drugs could be used in combination as potential chemotherapy to suppress NETosis.

## Supplementary Materials

The following are available online at https://www.mdpi.com/2072-6694/11/9/1328/s1. Figure S1: To clarify the anthracyclines inhibitory effect in the NETosis data of base line (-ve control) and LPS with anthracyclines drug (at 240 min), were further normalized by baseline and only LPS-mediated DNA release considering as 100% DNA release respectively, Figure S2: To clarify the anthracyclines inhibitory effect in the NETosis data of PMA with anthracyclines drug (at 240 min; last time point), were further normalized by only PMA-mediated DNA release considering as 100% DNA release, Figure S3: To clarify the anthracyclines inhibitory effect in the NETosis data of A23187 and ionomycin with anthracyclines drug (at 240 min; last time point), were further normalized by only A23187 and ionomycin-mediated DNA release considering as 100% DNA release respectively, Figure S4: Anthracycline mediated NETosis suppression does not induce apoptosis. Neutrophils were incubated with anthracyclines and then induced to produce NETs using either media only (-ve control), PMA (25 nM) or A23187 (4 µM) and then fixed by 4% paraformaldehyde in 8-chamber slides.
