# Supplementary material for: Anthracyclines Suppress Both NADPH Oxidase- Dependent and -Independent NETosis in Human Neutrophils
Source: Cancers (Basel). 2019 Sep 7;11(9):1328. doi: 10.3390/cancers11091328 (PMC6770146; doi:10.3390/cancers11091328)
Supplement: Supplementary file 1 [file cancers-11-01328-s001.pdf]

cancers

# Supplementary Materials: Anthracyclines Suppress both NADPH Oxidase-Dependent and -Independent NETosis in Human Neutrophils

Meraj A. Khan, Adam D'Ovidio, Harvard Tran and Nades Palaniyar

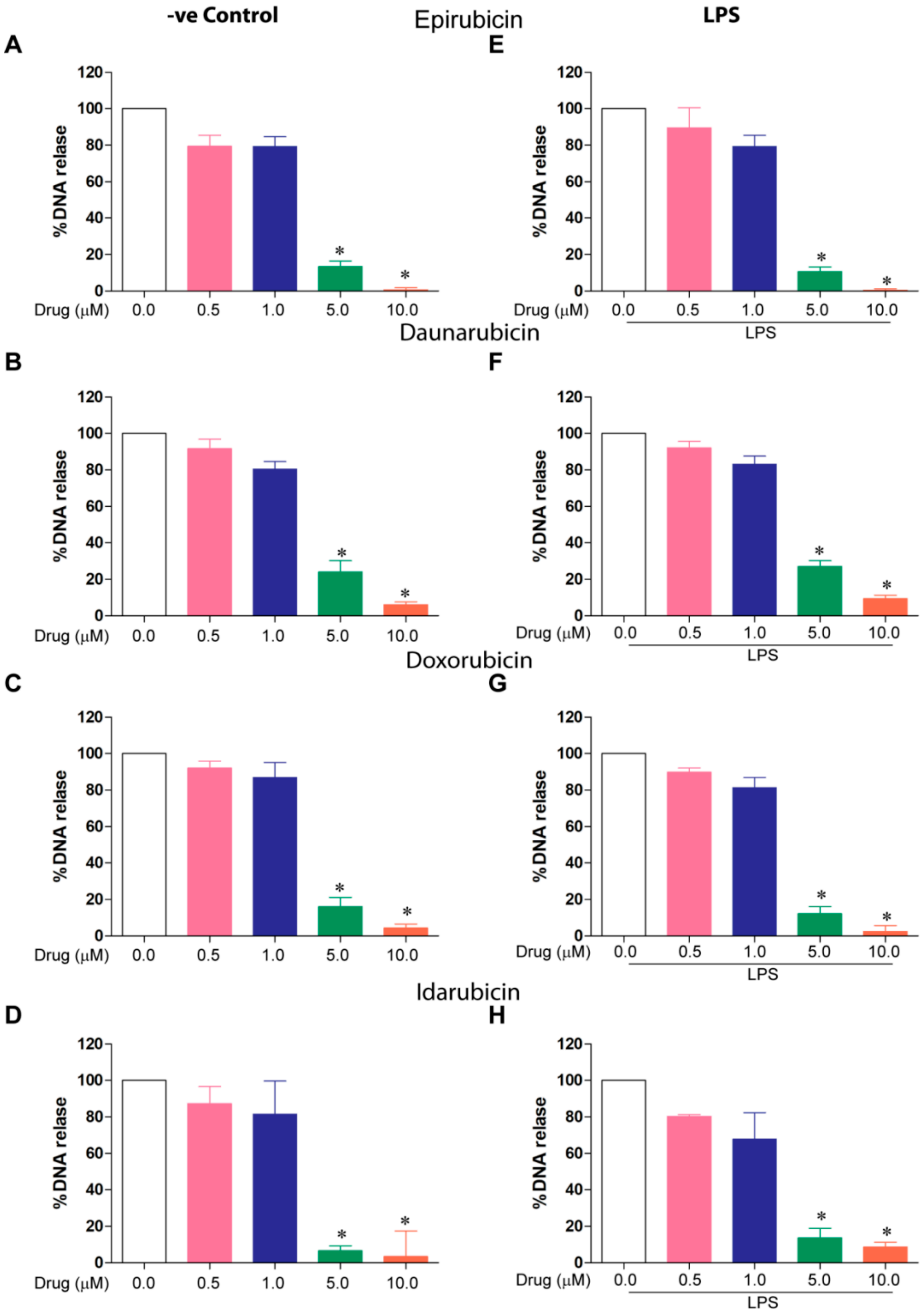


**Figure S1.** To clarify the inhibitory effect of anthracyclines in the NETosis data of base line (-ve control) and LPS with anthracyclines drug (at 240 min), were further normalized by baseline and LPS-mediated DNA release considering as 100% DNA release respectively. The bar graph shows the inhibitory effect of the drugs in baseline NETosis (**A**) Epirubicin, (**B**) Daunorubicin (**C**) Doxorubicin (**D**) Idarubicin and

in LPS mediated NETosis suppression by drugs (**E**) Epirubicin, (**F**) Daunorubicin (**G**) Doxorubicin (**H**) Idarubicin (* *p*-value < 0.05; One-sample *t* test, compares to hypothetical value 100).

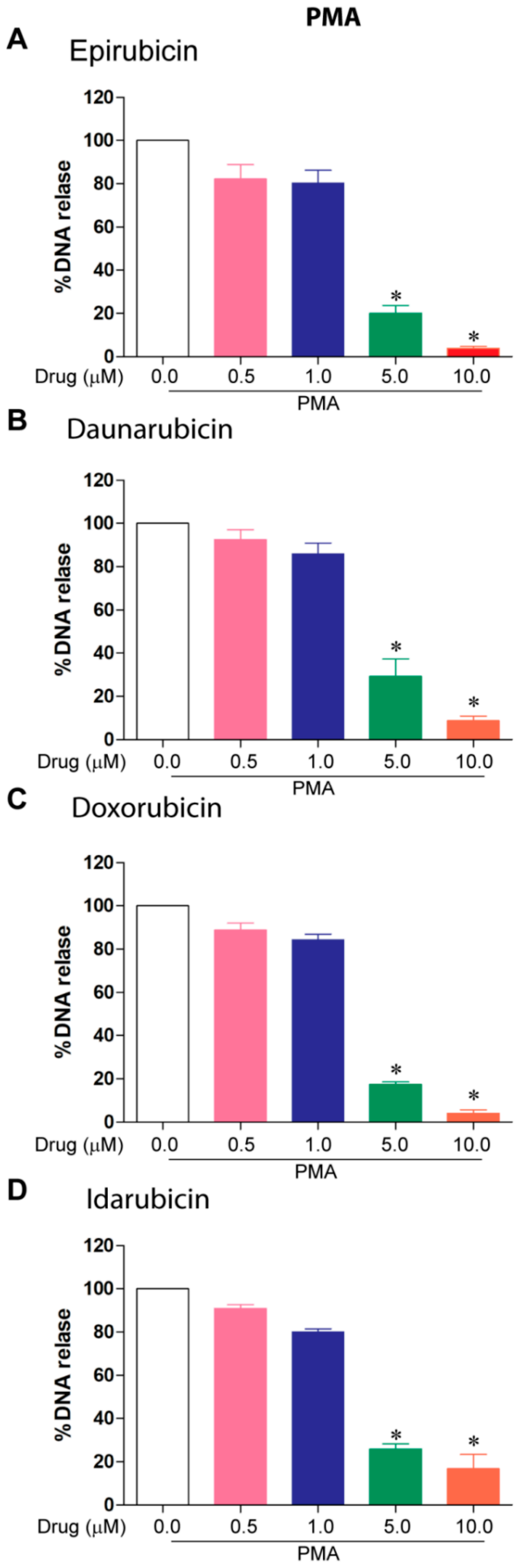


**Figure S2.** To clarify the anthracyclines' inhibitory effect in the NETosis data of PMA with anthracyclines drug (at 240 min; last time point), were further normalized by PMA-mediated DNA

release considering as 100% DNA release. The bar graph shows the inhibitory effect of the drugs in PMA mediated NETosis (**A**) Epirubicin, (**B**) Daunorubicin (**C**) Doxorubicin (**D**) Idarubicin (* $p < 0.05$; One-sample *t* test compared to hypothetical value 100).

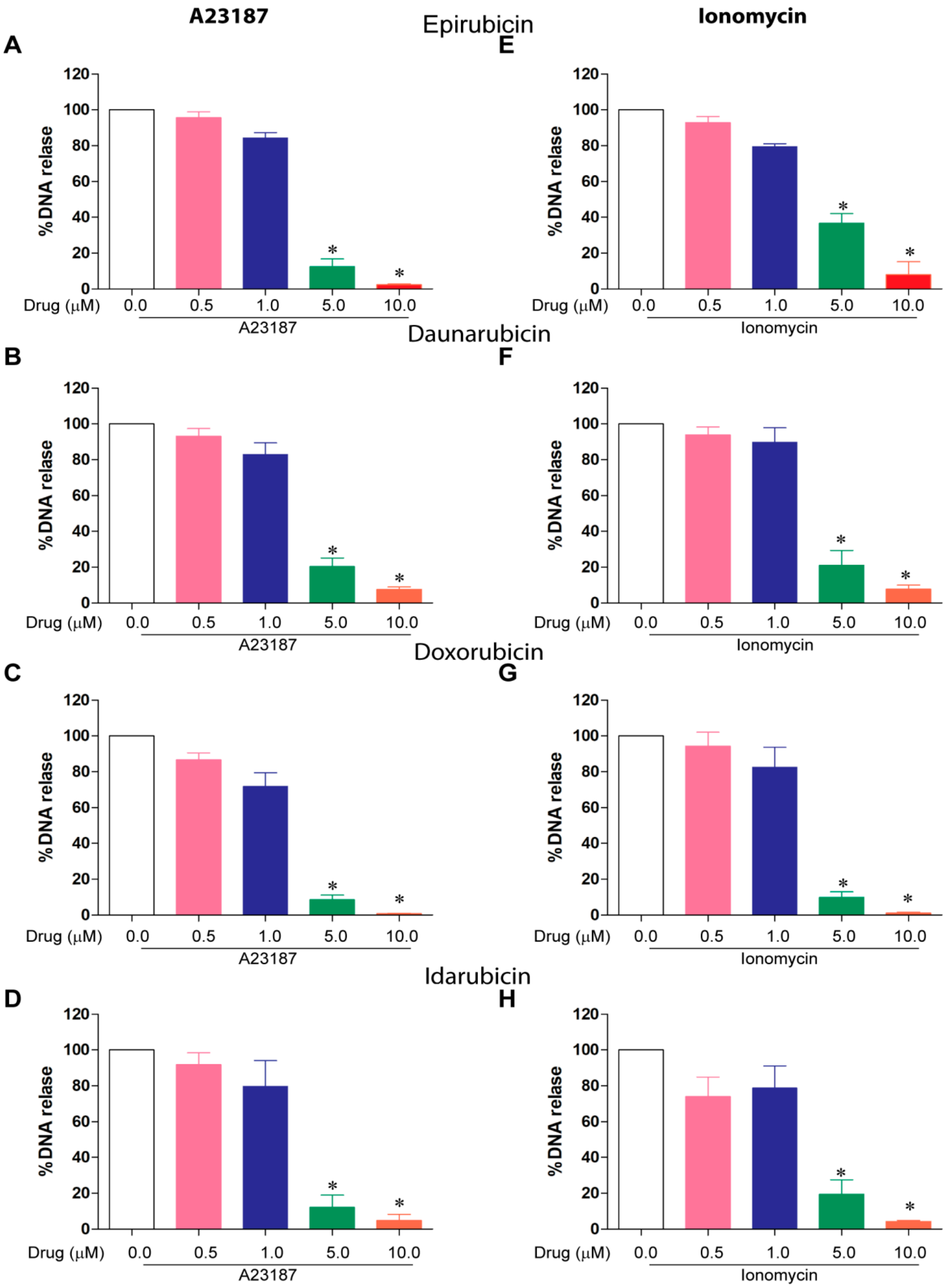


**Figure S3.** To clarify the anthracyclines' inhibitory effect in the NETosis data of A23187 and ionomycin with anthracyclines drug (at 240 min; last time point), were further normalized by A23187 and ionomycin-mediated DNA release considering as 100% DNA release respectively. The bar graph shows the inhibitory effect of the drugs in A23187-mediated NETosis (**A**) Epirubicin, (**B**) Daunorubicin (**C**) Doxorubicin (**D**) Idarubicin and in ionomycin mediated NETosis suppression by drugs (**E**) Epirubicin, (**F**) Daunorubicin (**G**) Doxorubicin (**H**) Idarubicin (* $p < 0.05$; One-sample *t* test, compared to hypothetical value 100).

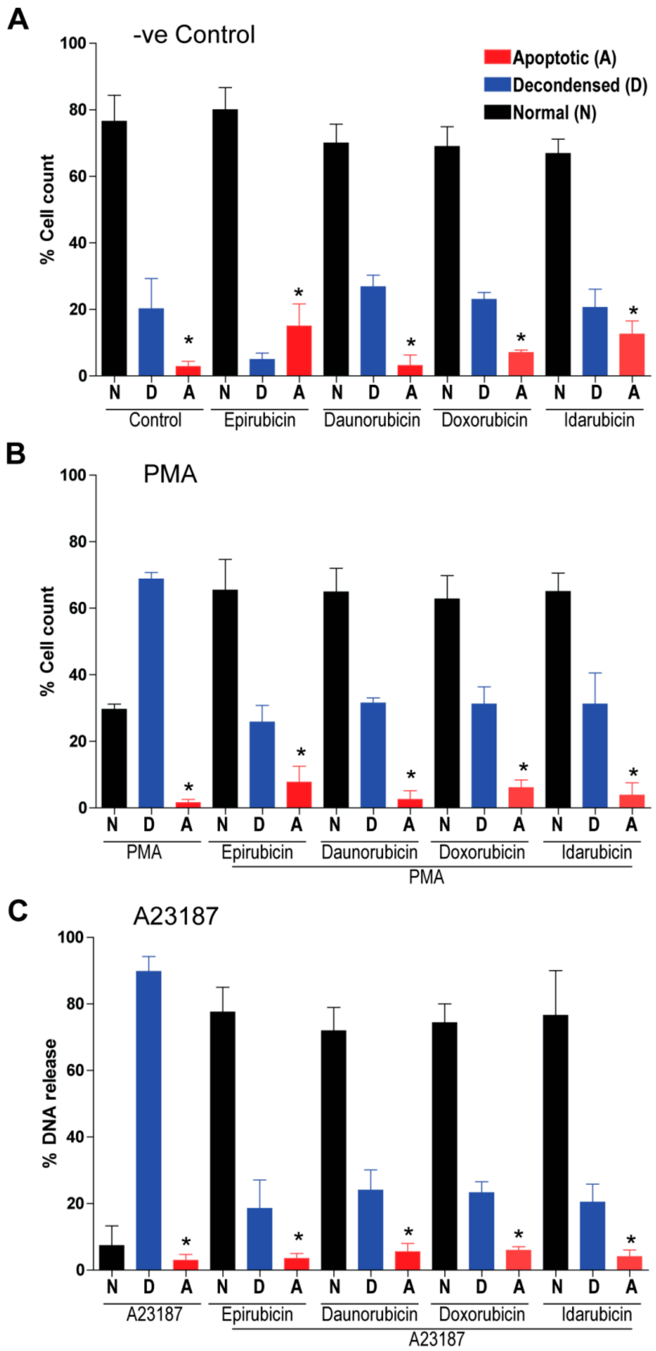


**Figure S4.** Anthracycline-mediated NETosis suppression does not induce apoptosis. Neutrophils were incubated with anthracyclines and then induced to produce NETs using either media only (-ve control), PMA (25 nM) or A23187 (4 µM) and then fixed by 4% paraformaldehyde in 8-chamber slides. cCasp-3 and DNA were stained with anti-cleaved cCasp-3 (red) and DAPI (blue). Based on the immunostaining and colocalization, cell and NETs counting data, show significantly reduced apoptosis in agonists and drug conditions compared to media control ($n$ = 3, * $p$-value < 0.05; One-way ANOVA with Tukey's post-test compared to apoptotic cells).

© 2019 by the authors. Licensee MDPI, Basel, Switzerland. This article is an open access article distributed under the terms and conditions of the Creative Commons Attribution (CC BY) license (http://creativecommons.org/licenses/by/4.0/).
